# Phase‐Change Solvents for Thermally Switchable Ion Conduction in Organogels

**DOI:** 10.1002/adma.202519014

**Published:** 2025-12-30

**Authors:** Yi‐ming Yuan, Thomas B. H. Schroeder

**Affiliations:** ^1^ Department of Textile Engineering, Chemistry and Science Wilson College of Textiles North Carolina State University 1020 Main Campus Drive Raleigh North Carolina USA

**Keywords:** electrolytes, ionotronics, organogels, phase‐change materials

## Abstract

Conductive media in which ions carry charge through a solution are foundationally important to batteries, supercapacitors, and ionotronic devices. Shifts in ion mobility imposed by physical changes in the solution can dynamically impact the conductivity of the medium. This paper reports a class of temperature‐responsive phase‐change organogels in which a polymer network is formed within salt solutions in organic solvents with melting points between room temperature and 100 °C. The conductivity in the molten state (up to ∼10^−4^ to 10^−3^ S/cm) exceeded that in the frozen state by over 10 000‐fold and remained stable after holding at 90 °C for 3 h or over 100 freezing/melting cycles. A diverse range of salts can be used, and the conductivity/temperature relationship can be tuned by selecting or mixing solvents with different melting points. Depending on the solvent composition, these phase‐change organogels can either produce approximately digital (binary) thermal responses to function as switches or respond continuously as analog temperature sensors or other transducers. An advantage of this strategy over prior literature is that the identity of the charge carrier and the phase behavior of the solvent can be tuned independently, presenting a wide design space for electrolyte materials whose conductivity responds to temperature.

## Graphical abstract

Organogels containing salts dissolved in phase‐change solvents exhibit thermally switchable ion conduction. The organogels, which display an over 10 000‐fold on‐off ratio and high cycling stability, can be synthesized with a wide range of salts and customizable conductivity/temperature relations for applications in ionotronics and energy storage. Demonstrations as both digital thermal switches and analog temperature sensors highlight the scheme's versatility.

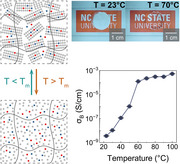

## 1 Introduction

Ionic conductors play a key role in several critical and emerging technologies. Notably, the ionic conductivity of electrolyte materials is a key factor toward determining the power output of batteries and supercapacitors [1, 2, 3]. In addition to these energy storage schemes, an expanding range of “ionotronic” devices in which ions serve as charge carriers in circuits alongside electrons includes sensors [4, 5, 6], actuators [7, 8, 9], and signal processing elements [10, 11, 12, 13]. The conductors in such devices often consist rather simply of well‐solvated ions in a liquid or gel, which have advantages over traditional metals and semiconductors – gels, for instance, are soft, transparent, and flexible, making them well‐suited to applications that (for example) involve interfacing with the body [14]. Using such solutions as conductors also means that a vast menu of solution‐phase processes – chemical reactions, phase behavior, transport phenomena, etc. – can be leveraged to influence and be influenced by the electrical signals being transduced.

In recent years, several reports have been published about materials that leverage crystallization/melting transitions to modulate the electrical impedance of ionic conductors over orders of magnitude [15, 16, 17, 18, 19, 20, 21, 22, 24, 25, 26, 27]. These schemes tend to employ electrolyte compounds that have melting points above room temperature but below 100 °C; the ions that compose each compound are mobile above the melting point and immobilized into crystal lattices below it. Many of these compounds have been salts in the imidazolium family, which are considered to be ionic liquids when in the molten state [22, 25, 28, 29]. Other reports describe polymer networks containing salt hydrates, several of which melt in this mild temperature range into high‐ionic‐strength aqueous solutions; these are often metastably supercoolable and require secondary nucleation [15, 17, 30]. Such materials with charge carriers that melt and crystallize have achieved high degrees of conductivity switching, with molten conductivities that exceed the frozen conductivities by up to ∼10^7^‐fold [25]. Such materials have been pitched as a means of locking in the charge of supercapacitors [22, 23, 25, 31] and as signal amplifiers [17], sensors [24, 26, 32] and smart devices [17, 20, 33].

A limitation of this approach, however, is that in each of these cases, the same chemical species is responsible both for being the charge carrier and for undergoing a phase change at the desired temperature. This greatly reduces the scope of the salts that can be employed in such materials, limiting the utility of these materials in energy storage applications where specific ions are required. It also limits the range of transition temperatures that can be selected, as selecting a particular temperature requires that a suitable electrolyte compound with the appropriate material compatibility and a melting point in the desired range can be found. Another class of recent papers sidesteps this limitation by relying on the melting and crystallization of side chains on the polymer network in a gel, but the conductivity ratio between states was typically more modest [24, 32].

**FIGURE 1:**
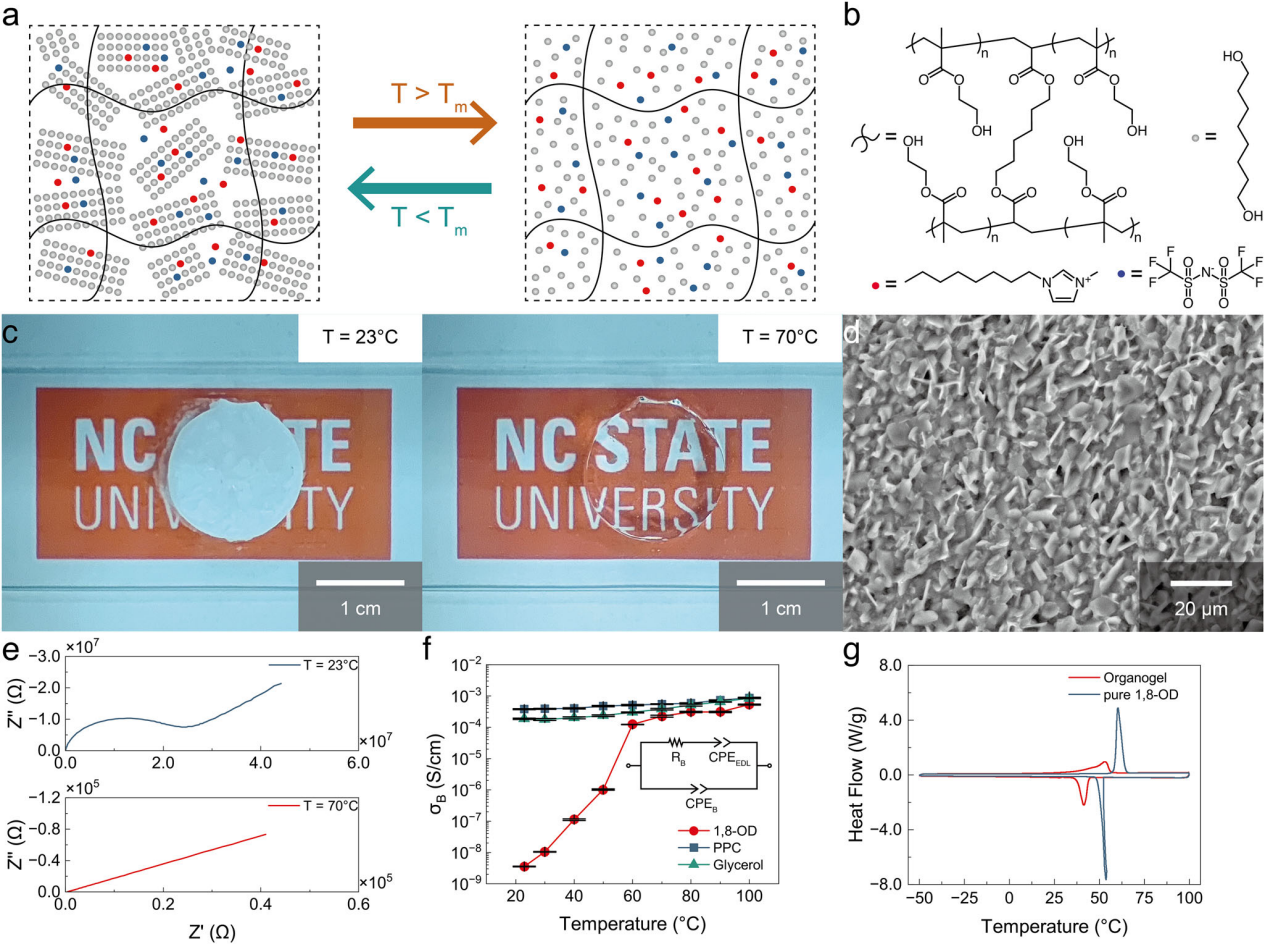
Principles and materials of the phase‐change organogel. Images and data presented here were taken from an organogel with a mass ratio of 40:50:10 (polymer:solvent:salt). a) Schematic of the working mechanism. b) Chemical structures of the constituents. The polymer network was poly(2‐hydroxyethyl methacrylate) cross‐linked by 1,6‐hexanediol diacrylate at a 200:1 (monomer:crosslinker) molar ratio, the phase‐change solvent was 1,8‐octanediol, and the salt was 1‐methyl‐3‐octylimidazolium bis(trifluoromethylsulfonyl)imide ([OMIM][TFSI]). c) Photographs of the organogel at 23 °C (left) and 70 °C (right). d) Scanning electron microscopy (SEM) image of the organogel (cross‐section) at room temperature. e) Nyquist plots of the organogel at 23 °C (top) and 70 °C (bottom). *Z’* and *Z”* (Ω) are the real and imaginary components of the impedance. f) Bulk ionic conductivity *σ_B_* as a function of temperature in organogels containing 1,8‐octanediol (1,8‐OD), a phase change solvent, as well as in gels containing the liquid solvents propylene carbonate (PPC) and glycerol. The inset shows the equivalent circuit model used for fitting the impedance data; *σ_B_* = *L*/(A*R_B_), where *L* (cm) is the thickness of the gel, *A* (cm^2^) is its cross‐sectional area, and *R_B_* (Ω) is the resistance as determined by fitting. CPE in the circuit model means “constant phase element.” Error bars in this paper denote standard error of the mean; sample size *N* = 6. g) Differential scanning calorimetry (DSC) traces of pure 1,8‐octanediol and the organogel (endotherm up and exothermic down, scan rate +5 °C/min).

Here we introduce organogels consisting of a polymer network solvated by one or more “phase‐change solvents” that exhibit a melting point in the desired range in addition to the salt that serves as the charge carrier (Figure 1a). At temperatures below its melting point, the solvent crystallizes within the polymer network. The resulting polycrystalline composite has a very low conductivity (10^−9^ – 10^−8^ S/cm), as the ions in the system are either trapped as inclusions inside solvent crystals or confined in the narrow interstitial gaps between them. Upon heating above the melting point, the crystalline domains melt, enabling the ions to migrate or diffuse through the liquid solvent; the ionic conductivity of the medium rises by orders of magnitude to up to 10^−4^ – 10^−3^ S/cm. We demonstrate that a large variety of salts can be employed as charge carriers in these systems and that phase‐change solvents can be selected or mixed to produce desired relationships between conductivity and temperature. The organogels exhibit excellent stability when held at a variety of temperatures or when the temperature is cycled repeatedly. Finally, we demonstrate the potential of these materials as customizable device components that can be formulated to behave either as digital, all‐or‐nothing switches or as analog sensors that respond continuously along a spectrum. These results establish clear design principles for creating high‐performance, programmable phase‐change ionic conductors, providing a materials platform for ionotronic devices and energy storage schemes in which temperature modulates conductivity.

## 2 Results and Discussion

Our model formulation (Figure 1b) employs a polymer network composed of 2‐hydroxyethyl methacrylate crosslinked with 1,6‐hexanediol diacrylate at a 200:1 monomer:crosslinker molar ratio, the phase‐change solvent 1,8‐octanediol (1,8‐OD), and the salt 1‐methyl‐3‐octylimidazolium bis(trifluoromethylsulfonyl)imide ([OMIM][TFSI]) as a charge carrier in a mass ratio of 40:50:10 (polymer:solvent:salt). We chose 1,8‐octanediol on the basis of its moderate melting point (60–62 °C), low volatility, low cost, benign safety profile, and the fact that it belongs to a family of similarly nonvolatile and benign di‐/polyols that have distinct melting points across the range from 20–100 °C and similar abilities to solvate a reasonably wide range of polymers and salts. The mixture was photopolymerized in the molten state at 80 °C.

The reversible phase transition is visually evident (Figure 1c). 1,8‐octanediol is a crystalline solid at room temperature (23 °C), so the bulk organogel is mechanically stiff and appears opaque due to light scattering from randomly oriented crystallites. When the organogel is heated to 70 °C (above the melting point), it becomes transparent and soft, reflecting the transition to a homogeneous phase with enhanced molecular mobility. In the crystalline state, 1,8‐octanediol forms crystals with a diameter of ∼5 µm (Figure 1d). The crystals are separated by thin channels at the grain boundaries that provide the only available pathways for ion transport in the crystalline state. In the molten state, liquid 1,8‐octanediol provides a continuous ion conduction pathway through the organogel.

We have performed electrochemical impedance spectroscopy (EIS) across a range of temperatures to quantify the relationship between the material's electrical properties and the temperature (Figure 1e). At room temperature (23 °C, the “off” state), the organogel exhibits extremely low conductivity (∼3.5 × 10^−9^ S/cm), confirming the effective suppression of ion transport. Upon heating to 70 °C (the “on” state), the conductivity increases sharply to ∼10^−4^ S/cm, a dramatic change compared to previously reported thermally responsive ionic conductors (Figure S1). The relationship between ionic conductivity and temperature correlates well with the phase change as detected by differential scanning calorimetry (DSC, Figure 1f). The most dramatic increase in conductivity occurs between 50 and 60 °C, coinciding with temperature range in which the bulk of the mixture changes phase. Above this range, the solvent is in the liquid state and the conductivity is relatively high. Below this range, the organogel occupies a largely crystalline, low‐conductivity state, though a tail in the phase transition between 20 and 50 °C is visible both in the conductivity plot (Figure 1e) and in the DSC trace (Figure 1g). The deviation from pure 1,8‐octanediol's melting point range of 60–62 °C is likely due to some combination of normal freezing‐point depression in mixtures and spatial heterogeneity effects – a heterogeneous population of crystallite sizes will have a range of effective melting temperatures due to the Gibbs‐Thomson effect, for example [34, 35, 36].

We arrived at the 40:50:10 polymer:solvent:salt mass ratio for this model system by measuring conductivity values as a function of temperature across the chemical space of the ternary mixture to maximize the on/off ratio (Figure S2a). Using the compounds chosen, formulations below 30 wt.% polymer did not cure into coherent, shape‐holding gels. Mixtures with low (and zero) solvent content were possible because [OMIM][TFSI] is a liquid at room temperature and could solvate the polymer network. Figure S2b presents the ionic conductivity at 23 °C (“off”) and 70 °C (“on”) as a function of [OMIM][TFSI] content at a fixed polymer content of 40 wt.%; the balance of the mixture was 1,8‐octanediol. As the mass percentage of [OMIM][TFSI] increased, both the “on” state and “off” state conductivities increased monotonically due to the greater availability of mobile ions. However, the “on” state conductivity increased steeply between 0 and 10 wt.% salt and subsequently plateaued at increasing salt concentrations, while the “off” state conductivity increased only mildly from 0 to 20 wt.% salt before increasing more steeply at higher salt concentrations, resulting in a pronounced maximum on/off ratio at a [OMIM][TFSI] mass percentage of 10%. This result suggests that at low salt concentrations, the melted state derives its conductivity from the quantity of mobile ions, while the crystallized state remains highly resistive due to effective ion trapping. At higher salt concentrations, the diminished amount of the crystallizing species resulted in a lesser degree of switching; thus, a low to moderate salt content is optimal for maximizing the switching performance. Likewise, Figure S2c presents the “on” and “off” state conductivities as a function of polymer content at a fixed [OMIM][TFSI] content of 10 wt.%. The on/off ratio exhibits a clear maximum at a polymer content of 40 wt.%, with a general trend that gels with an increasing polymer content (and thus decreasing solvent content) had a decreasing conductivity in the “on” state, reflecting lower ion mobility in the presence of less solvent. Increasing the polymer concentration also impacts the crystal size and morphology, as shown via scanning electron microscopy in (Figure S3).

**FIGURE 2:**
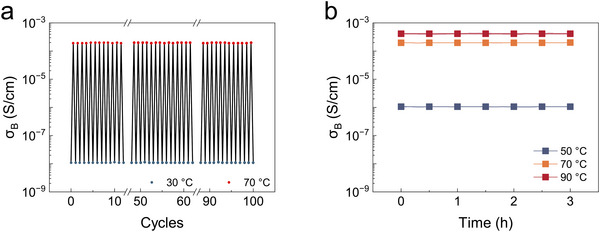
Stability of the phase‐change organogel. a) *σ_B_* remains approximately constant over several heating‐cooling cycles between 30 and 70 °C. b) *σ_B_* remains approximately constant a function of time when held at three different elevated temperatures (50, 70, and 90 °C).

We assessed the durability of the phase‐change organogel by subjecting it to 100 heating–cooling cycles between 30 and 70 °C. As illustrated in Figure 2a, the conductivity–temperature characteristics exhibited excellent stability throughout the cycles. The conductivity at specific temperatures remained nearly constant, indicating that the phase‐change mechanism was highly reversible and robust over repeated thermal cycling. At the same time, to investigate hysteresis in our system, we performed heating‐cooling cyclic tests on the phase‐change organogel between 30 and 100 °C in which the sample was allowed to equilibrate for a specific time (Figure S4a). The degree of hysteresis decreases as the equilibration time increases. It is easily rationalized that there is generally a time delay between the moment when a molten compound is brought below its melting point and the moment when crystals begin forming associated with the formation of critical crystal nuclei; the crystals must then grow to completion, which also takes time. At 50 °C the degree of undercooling (and thus the driving force toward crystallization) is low; accordingly, at this temperature, complete crystallization must take over 10 min, which leads to the disappearance of the hysteresis. We have additionally cycled the system ten times at a 5 min equilibration time; this test shows hysteresis loops with a stable shape (Figure S4b). Also, to evaluate the thermal stability of the conductive state, we held the organogel at 70 °C for 4 h and monitored its impedance (Figure 2b). The impedance remained nearly constant during this period, confirming that the organogel maintained its high‐conductivity state without significant degradation or drift, even under prolonged elevated temperature. Additionally, we have also shown that the temperature‐conductivity response of the organogel is unperturbed by exposure to humid air (Figure S5).

**FIGURE 3:**
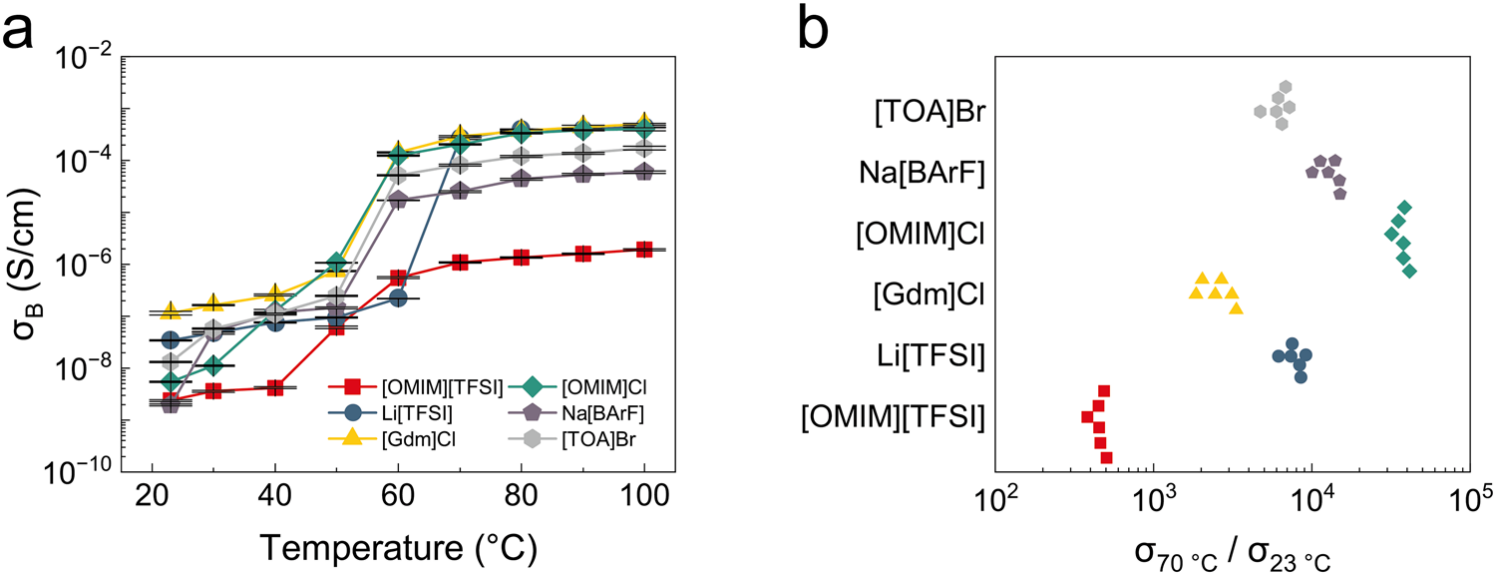
Universality of the phase‐change organogel. a) *σ_B_* of 1,8‐octanediol‐based organogels with different salts as a function of the temperature. Key: [OMIM][TFSI] = 1‐methyl‐3‐octylimidazolium bis(trifluoromethylsulfonyl)imide, [OMIM]Cl = 1‐methyl‐3‐octylimidazolium chloride, Li[TFSI] = lithium bis(trifluoromethylsulfonyl)imide, Na[BArF] = sodium tetrakis[3,5‐bis(trifluoromethyl)phenyl]borate, [Gdm]Cl = guanidinium chloride, [TOA]Br = tetraoctylammonium bromide. The polymer:solvent:salt mass ratio was 40:55:5. *N =* 6. b) On‐off ratios of organogels with different salts.

Some applications such as batteries may call for the use of particular ions in their electrolyte media. We hypothesized that phase‐change organogels operate by a mechanism that should be universally applicable across different salts, as long as a sufficiently good solvent for that salt with a melting point in the appropriate range can be found. We have validated this hypothesis by observing the conductivity of 1,8‐octanediol‐based organogels as a function of temperature for a wide range of chemically diverse salts: lithium bis(trifluoromethanesulfonyl)imide (Li[TFSI]), 1‐methyl‐3‐octylimidazolium chloride ([OMIM]Cl), guanidine hydrochloride ([Gdm]Cl), sodium tetrakis[3,5‐bis(trifluoromethyl)phenyl]borate (Na[BArF]), and tetraoctylammonium bromide ([TOA]Br) (Figure 3a). We used a mass ratio of 40:55:5 (polymer:solvent:salt) to ensure the formation of a homogeneous organogel for all species. Although multiple salts shown here are compatible with our system, it is worth emphasizing here that the salt must be well dissolved in the phase‐change solvent to form a homogeneous electrolyte solution above the solvent's melting point. The temperature responses of these organogels were similar in form (Figure 3a), each displaying a sharp increase in ionic conductivity around 60 °C that corresponds with the phase behavior of the solvent. All organogels show considerable on/off ratios (Figure 3b), which demonstrates that the concept of using phase‐change solvents to configure the ionic conductivities of organogels is general to a wide selection of salts. This consistency strongly demonstrates the universality of our phase‐change ionic conductor design. The underlying mechanism—ion trapping in the crystalline state and rapid release in the melted state—remains effective across a wide range of salts, as long as the salt dissolves and ionizes in the solvent.

The differences in conductivity/temperature relations between salts can potentially be attributed to a combination of structural differences and differences in interactions between the ions and the other components of the organogels. Conductivity will be determined at least in part by ionic radius, as ion mobility is inversely proportional to ionic radius per Stokes’ Law [37, 38]; this will apply at all temperatures but will perhaps be the overriding determinant at temperatures above the melting point. The three salts with the lowest conductivity at high temperatures are [OMIM][TFSI], Na[BArF], and [TOA]Br; Na[BArF] and [TOA]Br both are exceptionally large ions (MW > 400), whereas [OMIM][TFSI] is alone among the salts tested as consisting of two large ions (MW > 190). At temperatures at which the solvent is crystalline (< 60 °C), there are additional relevant parameters that may vary with molecular identity, such as i) the degree to which the ions are prone to inclusion in the growing solvent crystals vs exclusion into interstitial regions and ii) the degree to which the ions plasticize the poly(2‐hydroxyethyl methacrylate) polymer (which, when unplasticized, has a glass transition temperature from 85–105 °C) [39, 40, 41]. The complexity of these parameters puts disentangling them for each salt beyond the scope of this work.

The phase‐change solvent scheme allows engineers to specify the relationship between conductivity and temperature via the selection and mixture of solvent compounds. To demonstrate this, we incorporated different solvents with different melting points into gels containing [OMIM][TFSI] and plotted their conductivity–temperature profiles (Figure 3a). The additional solvents employed were 1,10‐decanediol (1,10‐DD, melting point 80–82 °C) and 1,2‐decanediol (1,2‐DD, melting point 40–42 °C); the mass ratio of all gels was 40:50:10 polymer:solvent mixture:salt. We have expressed the factor of conductivity change between incremental temperature measurements as Δlog(*σ_B_*)/Δ*T* (°C^−1^), where *σ_B_* (S/cm) is the conductivity, *T* is the temperature, and Δ represents the difference between measurements at adjacent temperatures (Figure 3b–d) – for example, the Δlog(*σ_B_*)/Δ*T* value comparing conductivity measurements at 30 and 40 °C was calculated as $\frac{\log\sigma_{B,\;40^{\circ }C}-\log\sigma_{B,\;30^{\circ }C}}{40^{\circ }C-30^{\circ }C}$ and plotted at the midpoint temperature, 35 °C. In each organogel, the conductivity changed by the largest factor in the temperature range that coincided with the largest endotherm measured by DSC. The literature melting point of the solvent was generally just above or at the high end of this temperature range (Figure S6) due to factors discussed previously (Figure 1g).

**FIGURE 4:**
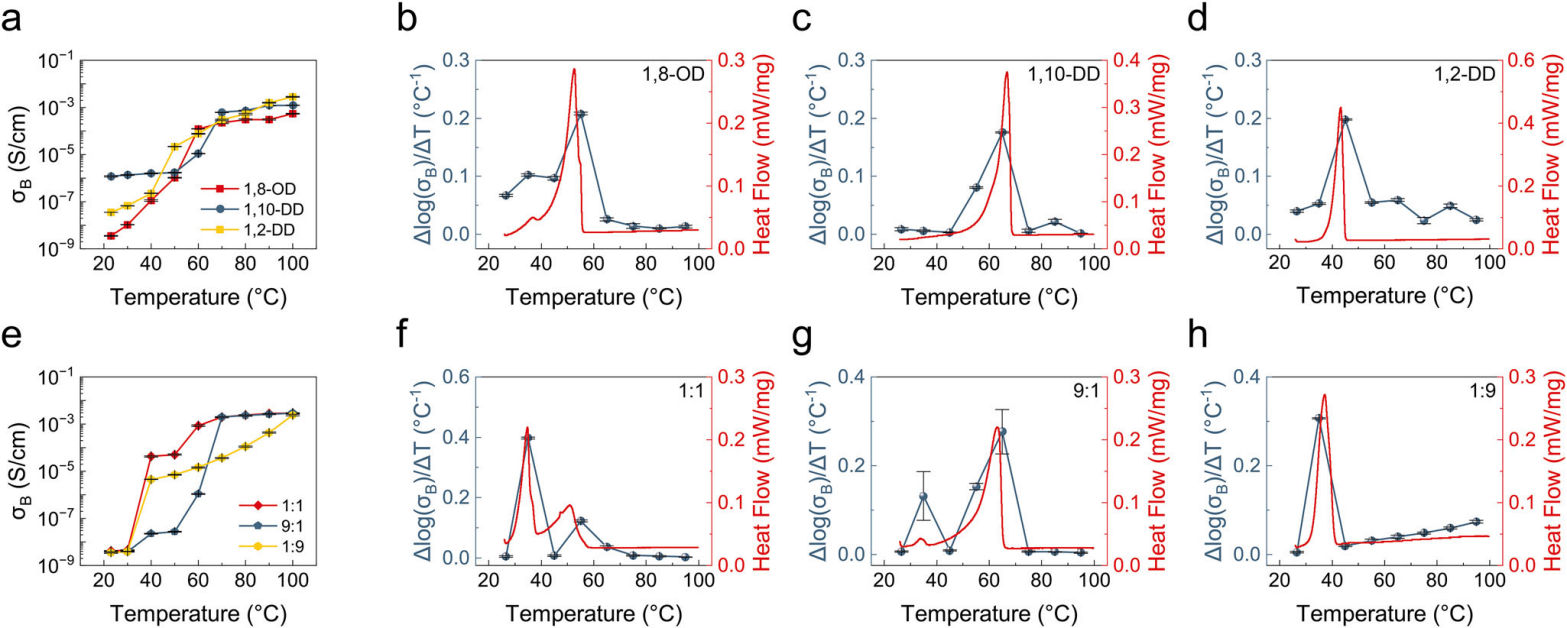
Relationships between the phase‐change properties and the thermal properties of the organogel. a) Conductivity (*σ_B_*) of organogels containing different solvents as a function of temperature. b–d) In a temperature series of *σ_B_* measurements, dividing the difference in log(*σ_B_*) by the difference in temperatures for each pair of adjacent temperatures results in a normalized expression of the factor by which *σ_B_* increases with increasing temperature. Δlog(*σ_B_*)/Δ*T* (°C^−1^) values calculated from the data shown in Figure 3a are plotted at the midpoint between temperatures compared and overlaid with DSC curves of organogels containing b) 1,8‐octanediol, c) 1,10‐decanediol and d) 1,2‐decanediol as the phase‐change solvent. e) *σ_B_* of the organogels containing 1,10‐decanediol:1,2‐decanediol mixtures at different ratios as a function of temperature. f–h) Δlog(*σ_B_*)/Δ*T* values calculated from the data shown in Figure 3e are plotted at the midpoint between temperatures compared and overlaid with DSC curves of organogels containing 1,10‐decanediol:1,2‐decanediol mixtures with ratios of f) 1:1, g) 9:1 and h) 1:9. All DSC curves are shown endotherm up and were measured with a with a scan rate of +1 °C/min. The mass ratio of all gels was 40:50:10 polymer:solvent mixture:salt. *N* = 6 for all measurements.

Next, we explored the effects of mixing two different phase‐change solvents—1,10‐decanediol and 1,2‐decanediol. We investigated three 1,10‐decanediol:1,2‐decanediol mass ratios, 1:1, 9:1, and 1:9 (Figure 4e–h). The conductivity–temperature plots for the 1:1 mixture in Figure 4e,f reveals two conductivity transitions in distinct temperature ranges, one between 30 and 40 °C (corresponding to 1,2‐decanediol) and one between 50 and 70 °C (corresponding to 1,10‐decanediol). The DSC curve in Figure 4f shows two endothermic melting peaks that correspond well to the temperature ranges of these transitions. For the mixture with a higher fraction of 1,10‐decanediol (Figure 4g), the melting peak and conductivity change in the higher temperature range were accordingly weighted more heavily. Surprisingly, for the mixture with a higher fraction of 1,2‐decanediol (Figure 4h), a single peak appeared instead of two separate peaks in both the Δlog(*σ_B_*)/Δ*T* plot and the DSC plot. Instead, after the peak at ∼36 °C corresponding to 1,2‐decanediol, both the conductivity and the slope of the DSC curve increased gradually and continuously; importantly, this effect appears in both types of measurement. Overall, by adjusting the composition of the solvents, the phase and conductivity changes can be configured to occur over a desired temperature window; these changes can occur either sharply, gradually, or at multiple transition points. This approach allows for customizable thermal gating of ionic conduction, expanding the versatility of phase‐change organogels for diverse applications with different requirements.

In all gels discussed in this work, there is some temperature‐dependence outside the phase change region. In order to rationalize this, we measured the relaxation properties of the gels shown in Figure 4b,h by sweeping the temperature from 25–100 °C using a dynamic mechanical analyzer in parallel plate shear mode at 10 rad/s at a 10^−4^ rad displacement (Figure S7). In both cases, the loss modulus decreases significantly at temperatures above the melting/freezing transition. This corresponds to a decrease in the viscosity of the medium with increasing temperature, which rationalizes both the positive ionic conductivity/temperature correlation seen in most gels in that regime and the fact that this increase is particularly great in the gel shown in Figure 4h, as the loss modulus decrease is highly pronounced in that gel (Figure S7b). Further, there is evidence that second‐order phase transitions may occur in such gels, shown most notably by a peak in the tan δ curve, which is often indicative of a glass transition.

**FIGURE 5:**
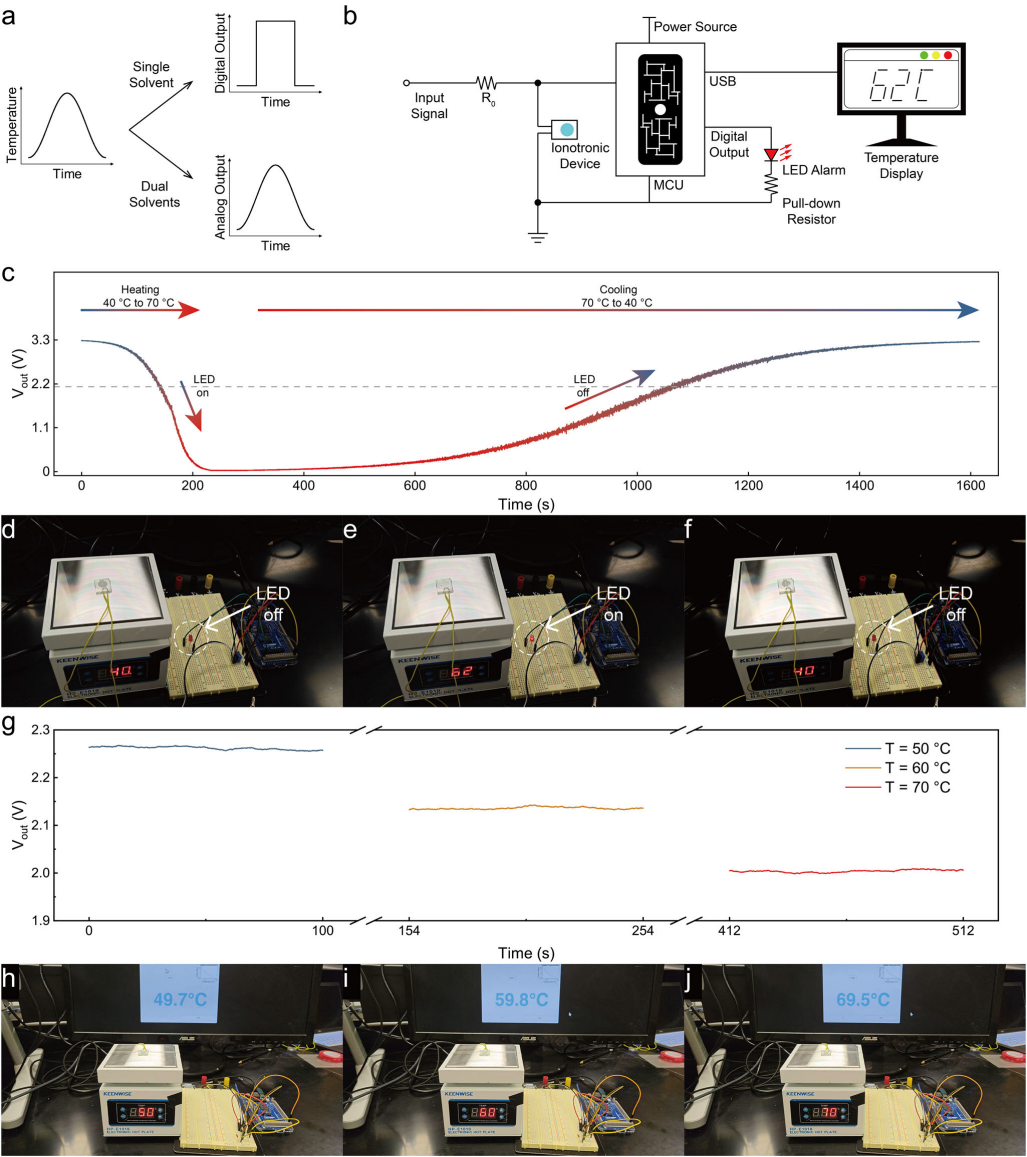
Demonstrations of phase‐change organogels in circuits. a) Illustration of theoretical output signals of organogels containing either a single solvent or a mixture of two solvents in response to a temperature stimulus. b) Schematic of the system containing the phase‐change ionotronic device, the output of which is directly connected via a microcontroller unit (MCU) to either an LED or a temperature display. The pull‐up resistor R_0_ was a potentiometer with a maximum resistance of 10 kΩ and was tuned before recording to optimize the system. The input signal was a square wave with a peak voltage of 3.3 V. c) The peak voltage across the thermal switch organogel plotted over time as the temperature rose from 40 to 70 °C, then fell back to 40 °C. d–f) Photographs of the thermal switch system when the temperature d) started below the threshold, e) rose above the threshold, and f) fell below the threshold (Movie S1). The threshold was set by tuning the potentiometer resistance to be approximately equivalent to the gel's resistance at the phase‐change temperature. g) Peak voltage across the temperature sensing organogel as a function of time at three holding temperatures (50, 60, and 70 °C). The pull‐up resistance R_0_ was fixed at 10 kΩ; the calibration procedure is shown in Movie S3. Photographs of the temperature sensing system when the temperature was h) 50 °C, i) 60 °C and j) 70 °C (Movie S2).

To show that the phase‐change organogels can be applied in various situations that require different temperature responses, we demonstrated that such gels can be configured to function in a similar manner to either digital switches or analog sensors, and can also be used to mitigate self‐discharge in energy storage devices. As illustrated in Figure 5a, conductors that undergo a dramatic change in conductivity at a specific temperature can be considered and applied as a temperature‐sensitive switch, while conductors with more gradual (and ideally more linear) conductivity‐temperature relationships may function as temperature sensors. To achieve an ionotronic thermal switch, an organogel with only one solvent (1,8‐octanediol) was sandwiched between two ITO‐glass electrodes (Figure S8) and connected to a microcontroller unit in a circuit with a pull‐up resistor, an input square wave signal and a signal reader (Figure 5b). The voltage across the organogel (peak voltage *V_out_* is shown in Figure 5c) could be connected to the microcontroller's digital input without signal post‐processing and act as a digital switch‐like control over (for example) a LED light (Figure 5d–f; Movie S1). To achieve an ionotronic temperature sensor, an organogel with a 1:9 mass ratio of 1,10‐decanediol:1,2‐decanediol (Figure 4h) was used due to its continuous resistance change with temperature above 40 °C. The voltage across the organogel at each temperature was stable (Figure 5g) and was interpreted by the microcontroller as a temperature (Figure 5h–j, Movie S2) after a voltage‐temperature calibration process in which the voltage was recorded at several temperatures for subsequent interpolation (Movie S3). In addition to this, we have demonstrated that as with previous reports of phase‐change electrolytes [22, 25], the phase‐change gels described here strongly inhibit self‐discharge processes in supercapacitors in the frozen state (Figure S9). In a demonstration 1,8‐octanediol‐based parallel‐plate supercapacitor charged to 1.0 V at 70 °C and held at open circuit, the voltage remaining at 5 h was 0.9 V when held at room temperature, whereas only 0.1 V remained when the system was held at 70 °C. Generally speaking, the phase‐change organogel can be deployed in systems with different requirements by configuring its constituents.

Several questions about phase‐change conductors remain that may serve as the subject matter of future reports; in particular, the determinants of conductivity in the “off” state seem complex. Ion conduction in the frozen state is assumed to take place mainly through the network of interstices between crystallites; however, the composition of the interstitial microenvironment cannot be readily assumed. The conductivity was observed to drastically decrease even prior to the nucleation of crystals in a freezing organogel (Movie S1), likely as a result of an increased solution viscosity at low temperatures; however, any mixture that occupies these interstices will be largely depleted of solvent (which forms the crystals). The degree of partitioning of additives, such as the ions in this system, between immobilization in the crystal lattice of a freezing phase‐change solvent and exclusion into the interstices will depend on the growth rate of the crystals [42, 43], which will in turn depend on the temperature and heat transfer properties of the environment. The phase‐change environment may also be manipulated to achieve alignment of crystals in (e.g.) the axial or radial direction, resulting in anisotropic conductivity [44]. As previously discussed, crystallite size affects both the geometry of the interstitial areas and the phase transition temperature and depends on the composition of the polymer network (Figure S3) These dependencies have not been explored in the present work and are worthy of future study.

## 3 Conclusion

In this work, we have extended the practical applicability of the phase‐change electrolyte scheme from the state of the art by expanding the palette of salts that can be employed and providing a straightforward means of tuning the conductivity/temperature relation. We have achieved this using a modular organogel formulation in which separate components carry out the function of undergoing a temperature‐mediated phase transition that modulates ion mobility (i.e. the solvent) and the function of carrying charge (the salt). These components can be swapped out as desired for particular applications, which may include ionotronic devices or the reduction of self‐discharge in energy storage devices that are designed for use at elevated temperatures and/or at infrequent intervals [22, 45, 46].

## 4 Experimental Section

### 4.1 Materials

2‐Hydroxyethyl methacrylate (HEMA, >97%, Sigma‐Aldrich), 1,6‐hexanediol diacrylate (HDDA, >99%, Fisher), lithium phenyl‐2,4,6‐trimethylbenzoylphosphinate (LAP, >97%, Ambeed), 1,8‐octanediol (1,8‐OD, 98%, Fisher), 1,10‐decanediol (1,10‐DD, >99%, Fisher), 1,2‐decanediol (1,2‐DD, >99%, TCI America), 1‐methyl‐3‐n‐octylimidazolium bis(trifluoromethanesulfonyl)imide ([OMIM][TFSI], >99%, Fisher), lithium bis(trifluoromethanesulfonyl)imide (Li[TFSI], >99%, TCI America), guanidine hydrochloride ([Gdm]Cl, >99%, Fisher), 1‐methyl‐3‐n‐octylimidazolium chloride ([OMIM]Cl, >97%, Fisher), sodium tetrakis[3,5‐bis(trifluoromethyl)phenyl]‐borate (Na[BArF], >97%, Matrix Scientific), tetra‐n‐octylammonium bromide ([TOA]Br, >98%, TCI America) were all used as purchased. Deionized water was generated by a Direct‐Q 3 UV system (MilliporeSigma).

### 4.2 Preparation of Phase‐Change Organogel

To prepare polymer precursor solutions, 10.41 g HEMA, 89.6 µL HDDA, 23.7 mg LAP, and 2 mL DI water were mixed thoroughly in a glass vial using a vortex mixer until the solution became transparent and homogeneous without any evident phase separation. This precursor solution was then mixed with one or more phase‐change solvents (e.g. 1,8‐octanediol) and salts (e.g. [OMIM][TFSI]) at the desired ratio and placed in an oven at 80 °C for 2 h, until the solution became transparent and homogeneous. The solution was injected while still hot into a disk‐shaped mold with a diameter of 11 mm, a thickness of 0.65 mm, and a transparent top and bottom. The filled mold was kept at 80 °C using a hot plate and exposed to UV light for 30 min. The cured organogel was dried in the oven at 80 °C overnight in order to remove the water.

### 4.3 Electrical Characterization

The organogel described above was sandwiched between two stainless steel electrodes covering the full area of the top and bottom surfaces and then assembled in a standard CR2032 coin cell. The sample was connected to a Gamry Reference 600 potentiostat/galvanostat with an oven for temperature control for electrochemical impedance spectroscopy in a 2‐electrode configuration at multiple temperatures. The frequency was scanned from 1 MHz to 1 Hz with 151 frequency points in total. The impedance data were collected and fit using an extended Debye circuit model (Figure 1f, inset) using MATLAB. *R_B_* in this circuit is considered to be the resistance of the sample. The conductivity (*σ_B_*) of the organogel was calculated as *σ_B_* = 4*L*/(*R_B_* ·π*d* ^2^), where *L* is the thickness of the sample and *d* is its diameter. When collecting data for the conductivity/temperature plots shown in the figures, the impedance was measured first at room temperature (23 C) and then at a series of increasing temperatures. Samples were generally equilibrated for 5 min after the oven reached its holding temperature before measurement unless otherwise specified. Linear sweep voltammetry was performed at +10 mV/s. For the measurement in humid air in Figure S5, the organogel was sandwiched between two stainless‐steel electrodes without further assembly to be exposed to the air and put into a closed temperature‐control chamber containing a beaker of water. Electrochemical impedance spectra were measured when the sample was initially raised to 30 °C at 0 h, after the temperature was raised to 70 °C soon thereafter, after the sample had been incubated in humid air at 70 °C for 12 h, and finally after the sample was allowed to cool down to 30 °C.

### 4.4 Differential Scanning Calorimetry

DSC (TA Discovery DSC 250) was performed with nitrogen used as purge gas. 5–8 mg of each sample was ground into powder form and encapsulated in a hermetically sealed aluminum pan. The sample, together with a reference pan, was heated from 25 to 100 °C while measuring differential heat flow at a scan rate of +1 or ±5 °C/min.

### 4.5 Scanning Electron Microscopy

Images were acquired with a Hitachi TM4000 desktop SEM. Cross‐sections were performed with a razor blade.

### 4.6 Dynamic Mechanical Analysis

A TA Instruments HR 20 dynamic mechanical analyzer was used to perform temperature sweeps run in parallel plate shear mode with an angular frequency of 10 rad/s and an oscillation displacement of approximately 10^−4^ rad.

### 4.7 Demonstration of a Thermal Switch and a Temperature Sensor

The organogel was cut into a disk shape with a thickness of 0.5 mm and a diameter of 8 mm and sandwiched between two pieces of glass coated in conductive indium tin oxide (ITO), which were used as electrodes. The device was assembled and sealed using a double‐sided tape (3M  4910 VHB, thickness = 0.65 mm) as a spacer; the ITO layers were connected to the circuit shown in Figure 5a using copper wires. An Arduino evaluation board (Arduino ABX00063) was used to both generate the input signal and read the output signal. A red LED light was connected to a digital output of the circuit board to function as the alarm in the demonstration of the thermal switch. In the temperature sensing demonstration, the circuit board was connected to a computer through Universal Serial Bus (USB); the temperature of the device could be viewed on the monitor. The temperature sensing system was calibrated by a program (Movie S3) by gathering multiple discrete points of the system's voltage‐temperature relationship (with temperatures set by the user) after the output voltage reaches equilibrium, and the calibration curve was obtained via shape‐preserving piecewise cubic interpolation. Note that the device was heated by a hot plate with a digital display, and the temperature reported by the hot plate can also be seen in Figure 5 and supplemental videos as a reference.

### 4.8 Measurement of Supercapacitor Self‐Discharge Decay

A 650‐µm‐thick organogel film (40:50:10 polymer:1,8‐octanediol:[OMIM][TFSI] by mass) in the shape of a square with a side length of 4 cm was sandwiched between two pieces of ITO glass (sheet resistance ≤15 Ω/sq) as electrodes using a silicone spacer. The resulting full‐cell capacitive parallel‐plate supercapacitor (with an electrolyte loading of approximately 65 mg cm^−2^) was connected to a circuit using a copper current collector and charged at 1 V using a Korad KD3005P programmable DC power supply for ∼2 h. The open‐circuit voltage decay after the charging process was measured using a high‐impedance electrometer (Keithley 6517A) to characterize the kinetics of self‐discharge.

## Funding

Funding for this work was provided by the Department of Textile Engineering, Chemistry and Science at North Carolina State University.

## Conflicts of Interest

The authors declare no conflict of interest.

## Supporting information




**Supporting File 1**: adma71966‐sup‐0001‐SuppMat.docx

**Supporting File 2**: adma71966‐sup‐0002‐MovieS1‐S3.zip

## Data Availability

The data that support the findings of this study are available from the corresponding author upon reasonable request.
