# Supplementary material for: Phase‐Change Solvents for Thermally Switchable Ion Conduction in Organogels
Source: Adv Mater. 2025 Dec 30;38(10):e19014. doi: 10.1002/adma.202519014 (PMC12910548; doi:10.1002/adma.202519014)
Supplement: Supplementary file 1 — Supporting File 1: adma71966‐sup‐0001‐SuppMat.docx [file ADMA-38-e19014-s002.docx]

Supporting Information

Phase-Change Solvents for Thermally Switchable Ion Conduction in Organogels

Yi-ming Yuan and Thomas B. H. Schroeder*


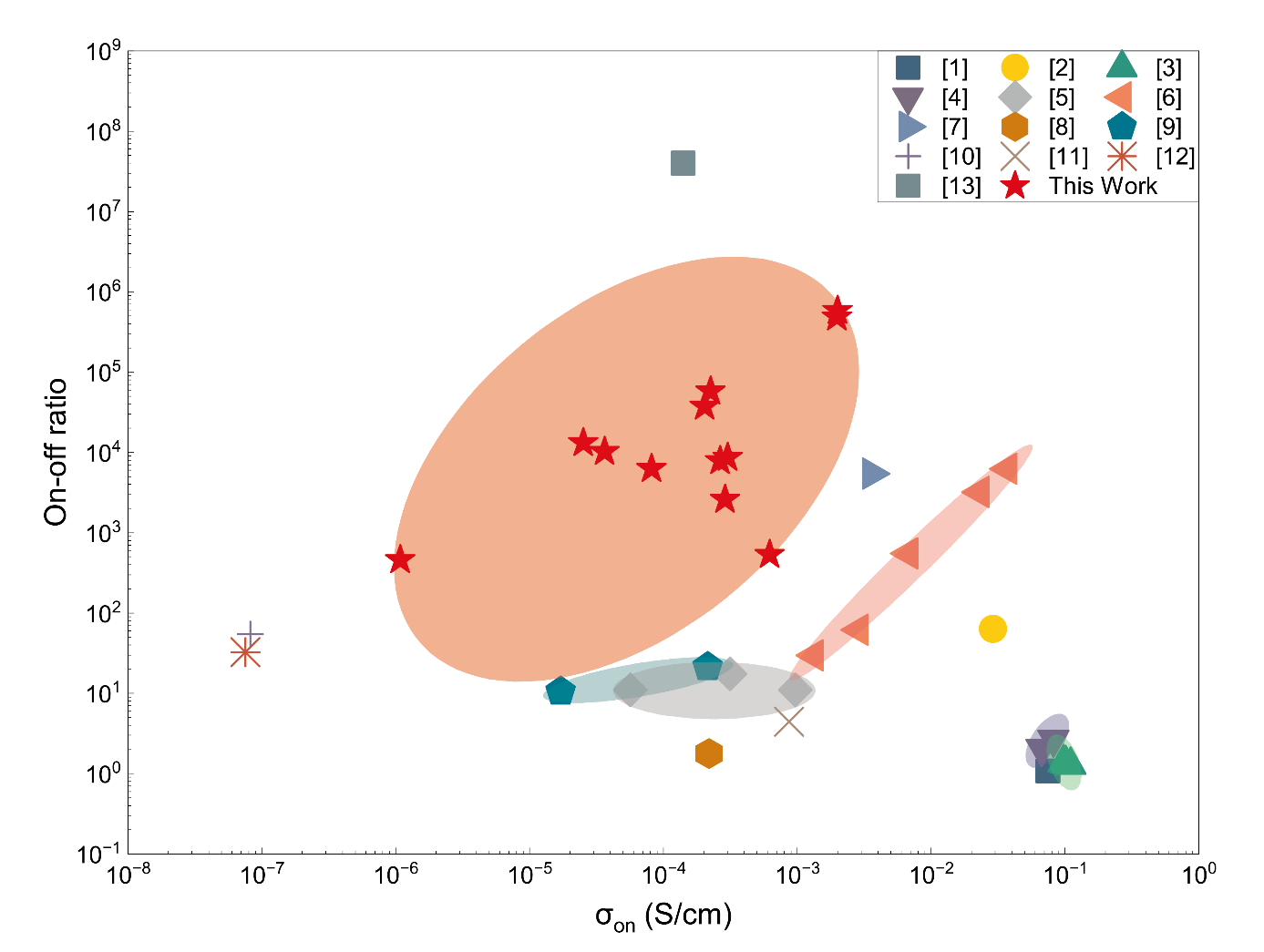


**Figure S1**. Comparisons among phase-change ionic conductors reported in prior literature and in this work along the axes of on-state conductivity (σ_on_) and on-off ratio.^[1-13]^


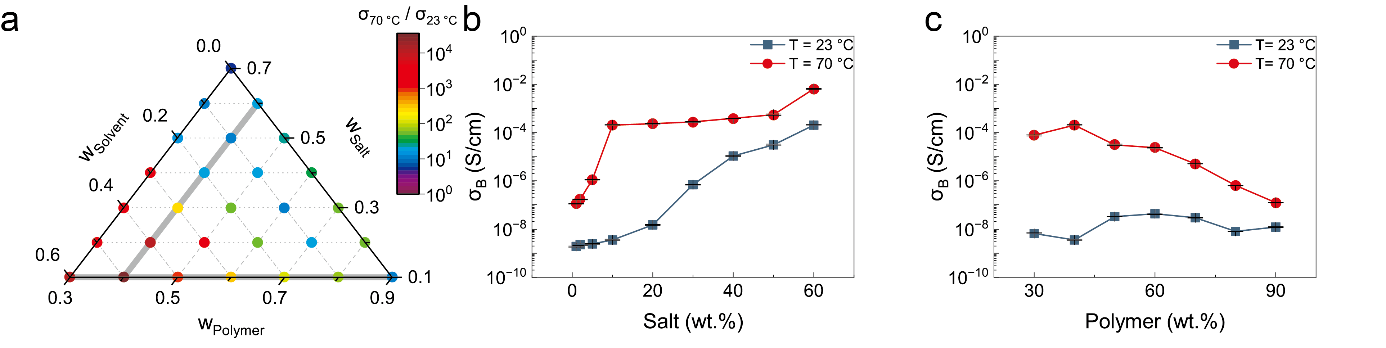


**Figure S2. Phase-change characteristics of the organogel with different ratios of the components.** a) Ternary plot of the on-off ratios (ratio between conductivity *σ_B_* at 70 °C and at 23 °C) with different mass fractions (*w_i_*) of the polymer network (poly(2-hydroxyethyl methacrylate) cross-linked with 1,6-hexanediol diacrylate at a 200:1 (monomer:crosslinker) molar ratio), the salt (1-methyl-3-octylimidazolium bis(trifluoromethylsulfonyl)imide, [OMIM][TFSI]), and the solvent (1,8-octanediol). The data points on the grey lines are shown in detail in b) and c). Conductivities at 23 °C and 70 °C as a function of the mass fraction of b) the salt (with polymer content fixed at 40 wt.%) and c) the polymer network (with salt content fixed at 10 wt.%). For all measurements, *N* = 6.


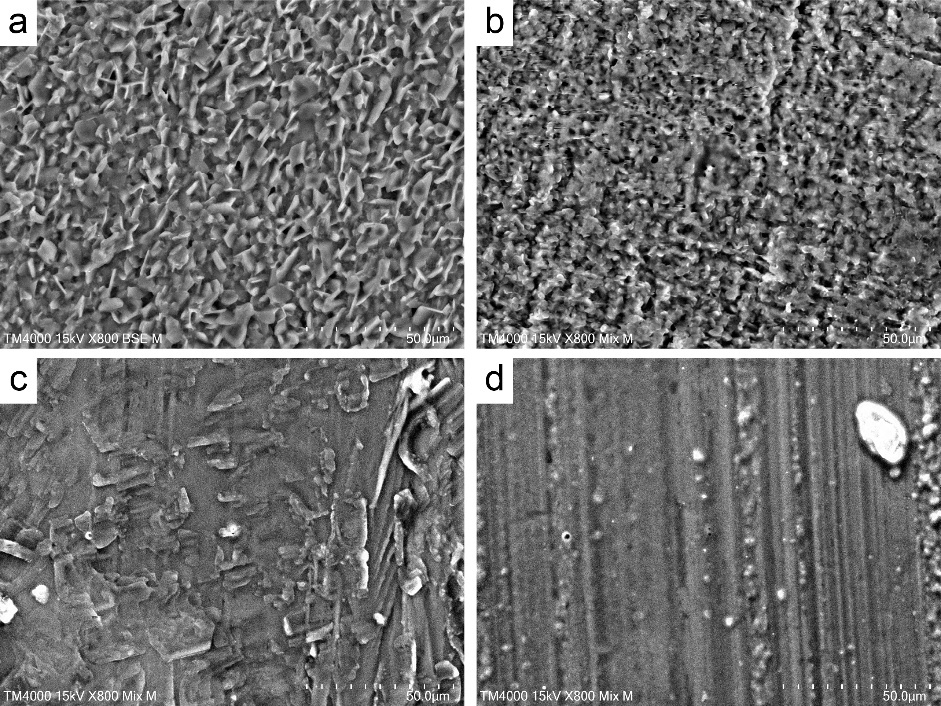


**Figure S3**. Cross-sectional scanning electron microscopic images of the organogels with the following mass ratios (polymer : 1,8-octanediol : [OMIM][TFSI]): (a) 40:50:10 (image repeated from **Figure 1d**), (b) 60:30:10, (c) 80:10:10, and (d) 90:0:10.


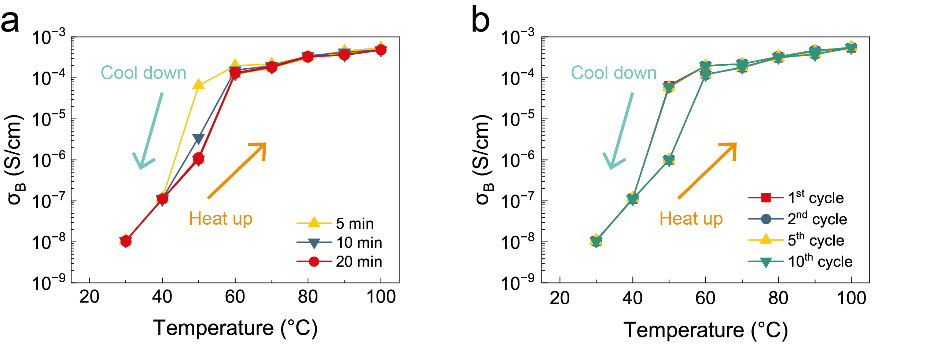


**Figure S4**. Hysteresis test of phase-change organogel. Relationship between ionic conductivity and temperature during thermal cycling up and down from room temperature (a) with equilibration times of 5 min, 10 min and 20 min at each temperature and (b) after up to 10 cycles with equilibration time of 5 min at each temperature.


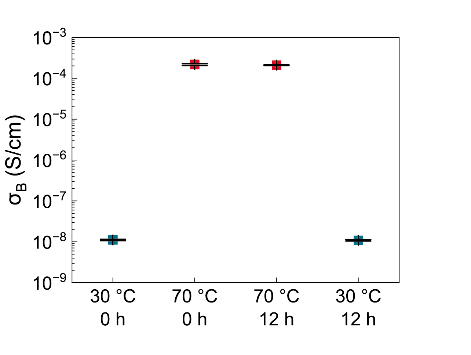


**Figure S5**. Ionic conductivity of the organogel at 30 °C (the first point), after being raised to 70 °C (the second point), after incubation for 12 h in humid air at 70 °C (the third point), and after cooling down to 30 °C in humid air (the fourth point). *N* = 6.


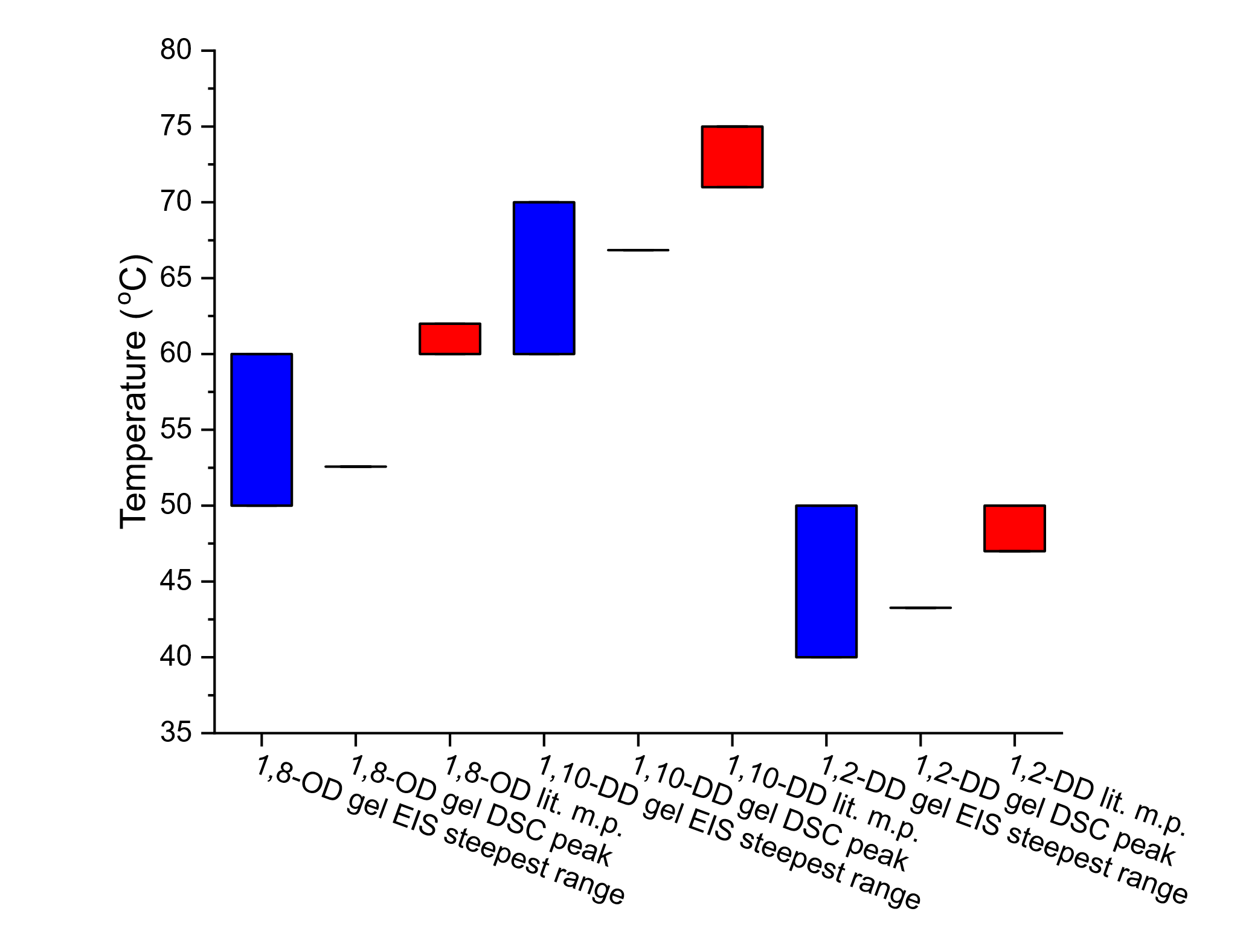


**Figure S6**. Comparison showing the following temperature ranges of significance for each of the three phase change gels formulated with different diols: the 10 °C interval in which the conductivity increased by the greatest factor with temperature (blue bars), the peak melting transition temperature as measured by DSC (lines), and the melting point range provided by the chemical supplier (TCI or Fisher).


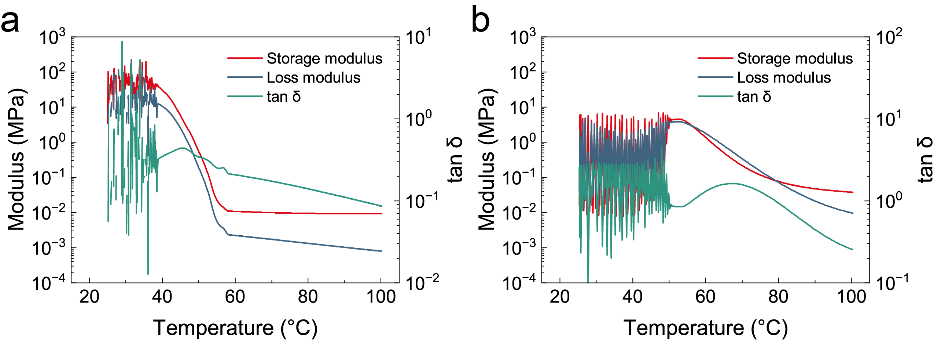


**Figure S7**. Dynamic Mechanical Analysis (DMA) of (a) the organogel in **Figure 4b** and (b) the organogel in **Figure 4h**. The DMA was run in parallel plate shear mode with an angular frequency of 10 rad/s and an oscillation displacement of approximately 10^-4^ rad.


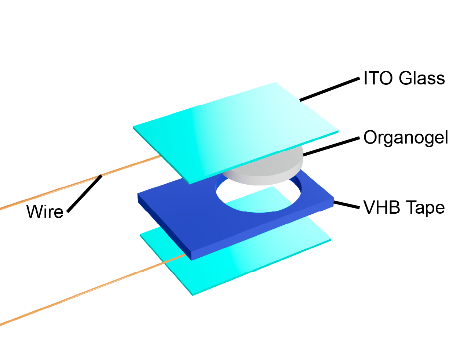


**Figure S8**. Structure of the ionotronic device in the demonstration of the organogel.


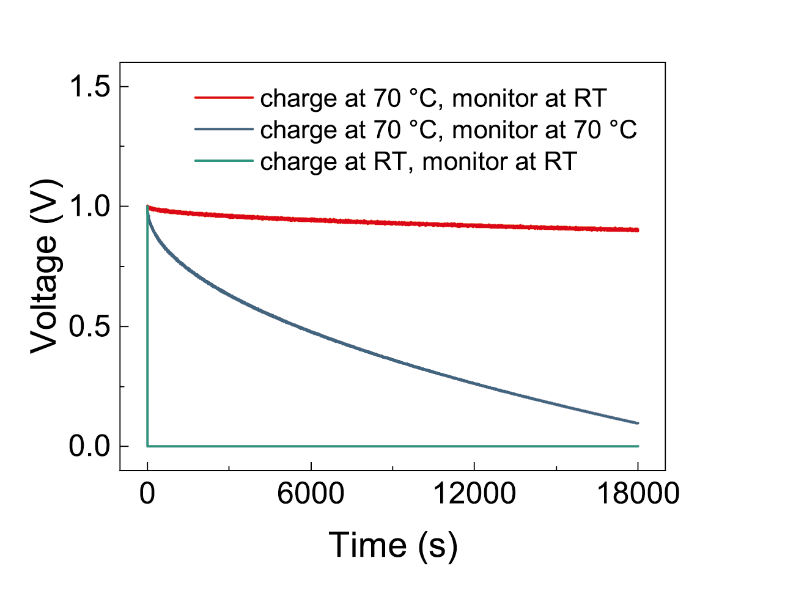


**Figure S9**. Demonstration of the organogel as a supercapacitor electrolyte with charging and charge decay at various temperatures. The charging voltage was 1 V, within the electrochemical window of the system (**Figure S10**), and the charging time was ~2 h for a fully charged case. The open-circuit voltage decay after the charging process was measured using a high-impedance electrometer (Keithley 6517A) to characterize the kinetics of self-discharge. A 650-μm-thick organogel was sandwiched with two pieces of ITO glass as electrodes, and the geometry of the electrode-electrolyte interface was a square with a 4-cm side length.


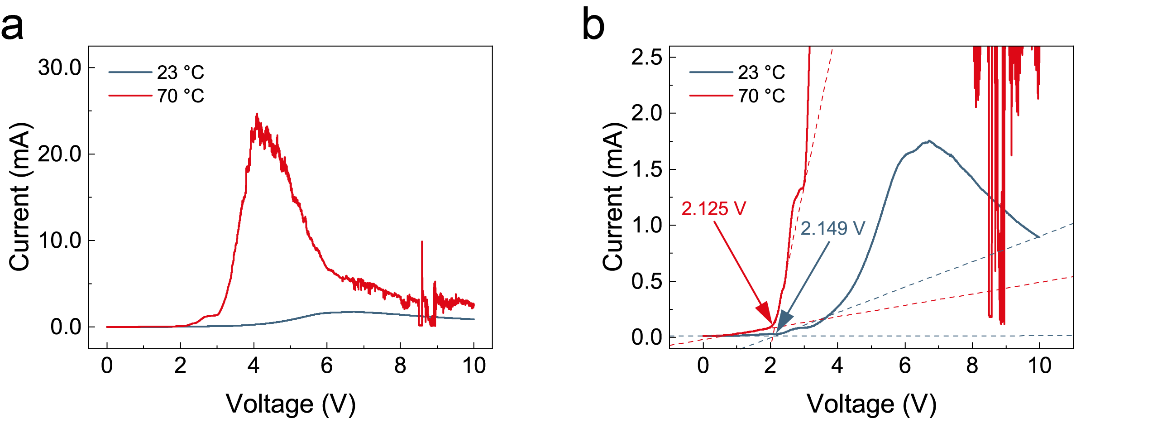


**Figure S10**. Current-voltage relationship of the organogel by linear sweep voltammetry (LSV). **Figure R6b** is a zoom-in version of **Figure R6a**. Highlighted values in **Figure R6b** are the electrochemical windows of the organogel at different temperatures. Scanning rate was 10 mV/s.

**References**

[1] Y. S. Yu, G. Huang, H. Y. Miao, J. H. Liang, X. Zhang, Y. Y. Liu, L. L. Tong, C. W. Dong, X. B. Fu, H. L. Huang, M. Ge, H. T. Liu, Y. Qian, *Acs Applied Energy Materials* **2025**, 8, 11706.

[2] H. Wang, S. Chen, X. Liu, Q. Zhang, X. Liu, *Journal of Energy Chemistry* **2025**, 100, 114.

[3] X. Shi, Y. Li, N. Shi, C. Ji, L. Hou, Y. Shi, J. Xu, Y. Lan, Q. Wei, G. Ma, P. Wu, Z. Hu, *Nat Commun* **2025**, 16, 9002.

[4] B. Niu, X. Jian, Z. Hu, P. Wang, X. Wang, *Adv Mater* **2025**, e14164.

[5] S. Shen, J. Li, Q. Wu, X. Chen, C. Ma, C. Liu, H. Liu, *Chem Commun (Camb)* **2024**, 60, 7363.

[6] X. Q. Ming, D. Zhang, H. Zhu, Q. Zhang, S. P. Zhu, *Adv. Funct. Mater.* **2024**, 34.

[7] R. Jia, X. Z. Duan, K. G. Wang, F. Q. Sun, T. Li, Z. Chen, L. Wang, G. Wang, L. W. Feng, H. D. Sun, M. F. Zhu, *Adv. Electron. Mater.* **2025**, 11, 2400408.

[8] X. Q. Ming, Y. F. Sheng, L. Yao, X. R. Li, Y. Y. Huang, H. Zhu, Q. Zhang, S. P. Zhu, *Chemical Engineering Journal* **2023**, 463, 142439.

[9] B. Yiming, Y. Han, Z. Han, X. Zhang, Y. Li, W. Lian, M. Zhang, J. Yin, T. Sun, Z. Wu, T. Li, J. Fu, Z. Jia, S. Qu, *Adv Mater* **2021**, 33, e2006111, 2006111.

[10] S. Park, B. Kim, C. Cho, E. Kim, *Journal of Materials Chemistry A* **2022**, 10, 13958.

[11] X. Ming, Y. Xiang, L. Yao, W. He, H. Zhu, Q. Zhang, S. Zhu, *ACS Appl Mater Interfaces* **2022**, 14, 47167.

[12] Y. T. Gong, Z. Z. Li, H. R. Li, W. Q. Wu, W. J. Zhou, J. Y. Zhao, C. G. He, M. Jiang, *Adv. Funct. Mater.* **2022**, 32.

[13] X. Q. Ming, L. Shi, H. Zhu, Q. Zhang, *Adv. Funct. Mater.* **2020**, 30.
